# An evaluation of a combined psychological and parenting intervention for HIV-positive women depressed in the perinatal period, to enhance child development and reduce maternal depression: study protocol for the Insika Yomama cluster randomised controlled trial

**DOI:** 10.1186/s13063-021-05672-0

**Published:** 2021-12-13

**Authors:** Tamsen J. Rochat, Samukelisiwe Dube, Kobus Herbst, Cecilia A. Hoegfeldt, Stephanie Redinger, Thandeka Khoza, Ruth Margret Bland, Linda Richter, Louise Linsell, Chris Desmond, Aisha K. Yousafzai, Michelle Craske, Ed Juszczak, Melanie Abas, Taygen Edwards, David Ekers, Alan Stein

**Affiliations:** 1 SAMRC/Wits Developmental Pathways for Health Research Unit, Faculty of Health Sciences, University of the Witwatersrand, Johannesburg, South Africa; 2 DSI-NRF Centre of Excellence in Human Development, University of the Witwatersrand, Johannesburg, South Africa; 3 Africa Health Research Institute, Durban, KwaZulu-Natal South Africa; 4 DSI-MRC South African Population Research Infrastructure Network (SAPRIN), Durban, South Africa; 5 Department of Psychiatry, University of Oxford, Oxford, UK; 6 Institute of Health and Wellbeing and Royal Hospital for Children, University of Glasgow, Glasgow, UK; 7 National Perinatal Epidemiology Unit, Nuffield Department of Population Health, University of Oxford, Oxford, UK; 8 Priceless, Faculty of Health Sciences, University of the Witwatersrand, Johannesburg, South Africa; 9 Harvard T.H. Chan School of Public Health, Boston, USA; 10 UCLA, Los Angeles, USA; 11 Institute of Psychiatry, Psychology and Neuroscience, King’s College London, London, UK; 12 Tees Esk and Wear Valleys NHS FT, Darlington, UK; 13 MRC/Wits Rural Public Health and Health Transitions Research Unit (Agincourt), School of Public Health, Faculty of Health Sciences, University of the Witwatersrand, Johannesburg, South Africa

**Keywords:** Perinatal depression, Behavioural activation, HIV, Parenting, Child development, Lay counsellors

## Abstract

**Background:**

The combination of poverty, HIV and depression in the perinatal period represents a major public health challenge in many Southern African countries. In some areas, up to a third of HIV-positive women experience perinatal depression. Perinatal depression is associated with negative effects on parenting and key domains of child development including cognitive, behavioural and growth, especially in socio-economically disadvantaged communities. Several studies have documented the benefits of psychological interventions for perinatal depression in low- and middle-income countries, but none have evaluated an integrated psychological and parenting intervention for HIV-positive women using task-sharing. This randomised controlled trial aims to evaluate the effect of a home-based intervention, combining a psychological treatment for depression and a parenting programme for perinatally depressed HIV-positive women.

**Methods:**

This study is a cluster randomised controlled trial, consisting of 48–60 geospatial clusters. A total of 528 pregnant HIV-positive women aged ≥ 16 years who meet the criteria for depression on the Edinburgh Postnatal Depression Scale (EPDS, score ≥ 9)) are recruited from antenatal clinics in rural KwaZulu-Natal, South Africa. The geospatial clusters are randomised on an allocation ratio of 1:1 to either the intervention or Enhanced Standard of Care (ESoC). The intervention group receives 10 home-based counselling sessions by a lay counsellor (4 antenatal and 6 postnatal sessions) and a booster session at 16 months. The intervention combines behavioural activation for depression with a parenting programme, adapted from the UNICEF/WHO Care for Child Development programme. The ESoC group receives two antenatal and two postnatal counselling support and advice telephone calls.

In addition, measures have been taken to enhance the routine standard of care.

The co-primary outcomes are child cognitive development at 24 months assessed on the cognitive subscale of the Bayley Scales of Infant Development-Third Edition and maternal depression at 12 months measured by the EPDS.

**Analysis:**

The primary analysis will be a modified intention-to-treat analysis. The primary outcomes will be analysed using mixed-effects linear regression.

**Discussion:**

If this treatment is successful, policymakers could use this model of mental healthcare delivered by lay counsellors within HIV treatment programmes to provide more comprehensive services for families affected by HIV.

**Trial registration:**

ISRCTN registry #11284870 (14/11/2017) and SANCTR DOH-27-102020-9097 (17/11/2017).

## Administrative information

**Table:** 

Title {1}	An evaluation of a combined psychological and parenting intervention for HIV-positive women depressed in the perinatal period, to enhance child development and reduce maternal depression: Study Protocol for the Insika Yomama Cluster Randomised Controlled Trial
Trial registration {2a and 2b}.	ISRCTN registry #11284870 and SANCTR DOH-27- 102020-9097.
Protocol version {3}	V1.05 26^th^ of May 2021
Funding {4}	The study is funded by the Joint Global Health Trials (MRC(UK)/DFID/Wellcome) [Grant number MR/P006965/1]. For the purpose of open access, the author has applied a CC BY public copyright licence to any Author Accepted Manuscript version arising from this submission. An additional supplement has been provided by the UKRI (UKRI COVID Allocation). The Oxford Health Biomedical Research Centre (BRC) has also provided some supplemental funding to the trial for the development of the electronic version of the therapy manual.
Author details {5a}	^1^ SAMRC/Wits Developmental Pathways for Health Research Unit, Faculty of Health Sciences, University of the Witwatersrand, South Africa ^2^ Africa Health Research Institute, KwaZulu-Natal, South Africa ^3^ DSI-MRC South African Population Research Infrastructure Network (SAPRIN), Durban, South Africa ^4^ Department of Psychiatry, University of Oxford, UK ^5^ Institute of Health and Wellbeing and Royal Hospital for Children, University of Glasgow, UK ^6^ DSI-NRF Centre of Excellence in Human Development, University of the Witwatersrand, Johannesburg, South Africa ^7^ National Perinatal Epidemiology Unit, Nuffield Department of Population Health, University of Oxford ^8^ Priceless, Faculty of Health Sciences, University of the Witwatersrand, Johannesburg, South Africa. ^9^ Harvard T.H. Chan School of Public Health, Boston, USA ^10^ UCLA, USA ^11^ Institute of Psychiatry, Psychology and Neuroscience, King’s College London ^12^ Tees Esk and Wear Valleys NHS FT ^13^ MRC/Wits Rural Public Health and Health Transitions Research Unit (Agincourt), School of Public Health, Faculty of Health Sciences, University of the Witwatersrand, Johannesburg.
Name and contact information for the trial sponsor {5b}	University of Oxford Research Services, University Offices Wellington Square, Oxford OX1 2JD Tel. + 44 (0) 1865 282585 E-mail: oxtrec@admin.ox.ac.uk
Role of sponsor {5c}	The funder had no role in the design of this study and will not have any role during its execution, analyses, interpretation of the data, or decision to submit results.

## Introduction

### Background and rationale {6a}

#### Perinatal depression in the context of HIV

Perinatal depression is common amongst HIV-positive women globally and a major public health challenge [1, 2]. A 2015 systematic review of African studies of perinatal depression amongst HIV-positive women reported rates between 23.4 and 43.5% in the antenatal and 22.5 and 31% in the postnatal periods [3]. More recently, a global meta-analysis comparing rates of depression amongst HIV-positive and HIV-negative women in both the antenatal and postnatal periods, found that HIV-positive women had significantly higher odds of depressive symptoms [2]; 36% of HIV-positive women experienced antenatal depression compared to 26% of HIV-negative women, whilst 21% of HIV-positive women experienced postnatal depression compared to 16% of HIV-negative women. Given that up to a third of all women attending antenatal services in Southern Africa are HIV-positive, these differences are clinically important.

Perinatal depression may contribute both directly and indirectly to a wide range of maternal and child health risks [4, 5]. Studies show that perinatal depression amongst HIV-positive women is associated with increased suicidal ideation, with rates as high as 40% in some studies [3, 6]. Amongst HIV-positive women, perinatal depression has been associated with lower clinic attendance and poor adherence to antiretroviral treatment (ART) [7, 8]. A recent systematic review and meta-analysis estimated that, for people living in sub-Saharan Africa, depression approximately doubled the odds of non-adherence (OR 2.54 95% CI [1.7-3.9]) [9]. Psychological therapies, especially those using cognitive behavioural approaches have been shown to improve both depression and adherence to ART in the USA [10, 11], although more evidence is needed in African countries [12].

The potential impact of perinatal depression on parenting and child development is of major concern. Studies have consistently shown that perinatal depression is associated with negative effects on children’s cognitive, language, behavioural and emotional development as well as growth, including risks of stunting [5, 13]. The persistence of depression appears to be more likely to lead to negative effects on the child [14], highlighting the need for treatment. The quality of caregiving is a key mediator of the negative effects of parental depression on child development. An important reason for this is the effects of rumination, a core characteristic of cognition in depression, on responsiveness to a young child [15]. Rumination consists of recurrent negative thoughts that are intrusive and difficult to dismiss, absorb attention and are associated with reduced problem-solving, speed of response to external stimuli and disturbances in attention. This is important because focused maternal attention to the infant and contingent responsiveness to infant cues and behaviour is essential to early cognitive and emotional childhood development. Furthermore, depression disrupts the ability to scaffold and support an infant’s exploratory behaviour and emotional states, especially infant distress and this can disrupt emotional and behavioural development [5, 16].

Finally, maternal depression may affect child growth, gastrointestinal and respiratory infections, and general health through a shorter duration of exclusive breastfeeding (EBF) and lower rates of health and hygiene promoting behaviours [17, 18]. EBF is of particular importance in HIV-positive populations as studies have found that optimal early-life feeding practices ameliorate the effects of being born to an HIV-positive mother [19]. Furthermore, EBF has been shown to reduce episodes of diarrhoea in infants born to HIV-positive mothers [20].

Consequently, maternal depression in the context of HIV is associated with several maternal and child health risks. The combination of HIV and depression in the perinatal period is especially important because the negative impact of depression on children is amplified by socio-economic adversity and lack of support [4, 5], which are associated with HIV [21]. Thus, the clinical implications of perinatal depression amongst HIV-positive women are likely extensive in HIV-endemic regions.

These effects of depression in the context of HIV on child development forms part of a growing body of literature underscoring the importance of early-life exposures for long-term health and development [22–24]. As a result, international agencies, including the World Health Organization, are developing evidence-based policies and interventions aiming to improve early child development, encapsulated in the Nurturing Care Framework [25]. The Nurturing Care Framework has two guiding implementation features; firstly, to ensure the child receives multiple intervention inputs required to support healthy development, including adequate nutrition and health, early learning opportunities, safety and security and responsive care. Secondly, the framework attends to the enabling environment of care, including the mental health of caregivers and their support [24]. The present study is situated within this new scientific and policy focus.

#### Interventions for perinatal depression in LMIC

In recent years, task-shifting of mental health care services to trained lay health care workers has been strongly advocated for in an effort to increase treatment coverage for mental health disorders in LMICs [26, 27]. A range of intervention studies in LMICs have used task-shifting to community health workers (CHW) or lay counsellors to support women with perinatal depression. Internationally, such studies report that psychological treatment by peer or lay counsellors is feasible and generally has positive effects compared to routine standard of care [27]. A 2014 review of nine South African psycho-social interventions delivered by CHWs or lay counsellors (several of which targeted pregnant women) reported that the majority of studies provided evidence for the effectiveness of task-shifted interventions which include psychological content [28], although one recent RCT in South Africa for perinatal depression, not specifically targeting HIV-positive women, did not report significant effects on maternal mood [29].

There has been one example of a randomised controlled treatment trial (Masihambisane Trial) which tested the effectiveness of a clinic-based peer-mentor support intervention addressing health, including mental health, and stigma faced by perinatal HIV-positive women (not necessarily experiencing depression) in rural KwaZulu-Natal, South Africa. This study showed improvements in maternal mental health but also found that centre-based activities presented significant challenges to retention [30]. To our knowledge, only one published RCT in Africa has directly tested the effects of an intervention specifically for HIV-positive pregnant women on perinatal depressive symptoms (and prenatal disclosure rates of HIV). The trial compared a 6-week psycho-social support group facilitated by nursing staff with the standard of care (SoC) in Dar es Salam, Tanzania. The trial found that marginal non-significant improvements in the intervention arm compared to the SoC arm and there was a high attrition rate [31].

In addition to Kaaya et al. (2013) [31], other studies in LMICs have reported high attrition rates in clinic- and group-based studies exploring mental health interventions in HIV-positive populations. Petersen et al. reported an attrition rate higher than 45% in a randomised controlled pilot study of group-based interpersonal therapy intervention for depressed HIV-positive patients in South Africa [32]. The low uptake of the intervention was attributed to limited transport opportunities and money, employment responsibilities on treatment days and discomfort with the group approach (particularly gender and age similarities were cited as important for ensuring comfort in relaying sensitive issues). As mentioned above, the Masihambisane found low uptake of sessions in a clinic-based peer-mentor intervention for HIV-positive women in the perinatal period, citing opportunities and costs and clinic waiting times as significant barriers to clinic-based interventions [30]. In contrast, home-based interventions for HIV-positive women in South Africa have been documented to reach more than 98% of women and follow-up rates were more than 91% at 6 months [33]. Thus, in the present study, we planned to use a home-based individual intervention to overcome some of the barriers to clinic-based group interventions.

#### Interventions for parenting in LMIC

Parenting interventions that support caregivers and provide guidance on practices and skills that help caregivers to support their young child’s development have been beneficial in improving early childhood development (ECD) outcomes and reducing risks of developmental delay. The most recent global meta-analysis of parenting interventions, comprising 90 RCTs, found small (*β* = 0.19, socioemotional) to medium (*β* = 0.31–0.34, cognitive and language) effects on ECD. It is noteworthy that interventions that included strategies to support responsive caregiving had stronger effects on children’s development [34], however, there are evidence gaps. Firstly, only a small number of parenting interventions implemented in LMIC evaluated children’s early socioemotional development. Secondly, fewer interventions have assessed caregiver mental health. In a review that examined care outcomes for parenting interventions in LMIC, nine of the 15 interventions assessed maternal depressive symptoms and the meta-analyses did not report any significant reduction in depressive symptoms [35]. Parenting interventions that include components to promote mental health, rather than focusing only on the needs of the child, are likely to be beneficial for children and their caregivers [36]. More research is needed on how these interventions can be effectively combined and tailored to the needs of at-risk families.

To our knowledge, the present study is the first to test a home-based integrated intervention specifically targeting HIV-positive, perinatally depressed women, which combines treatment for depression with a parenting intervention delivered by lay counsellors.

#### The Insika Yomama intervention

In this trial, we test a novel integrated intervention to treat HIV-positive perinatally depressed women and enhance child development, using a combination and adaptation of two evidence-based interventions: (i) behavioural activation (BA) for depression and (ii) and the WHO/UNICEF Care for Child Development (CCD) package.

*Behavioural activation (BA)* is being trialled because it has been shown to be as effective as cognitive behavioural therapy (CBT) in high-income settings [37–39]. BA is easier to deliver than CBT since it does not require extensive training or complex counselling skills. Studies suggest that BA delivered by non-specialists appear similar to those with formal therapy qualifications [40]. Importantly, studies report that BA is effective in treating depression in LMIC when delivered by a range of non-specialist health care workers [41]. As a result, BA lends itself to task-shifting [42, 43]. In South Africa, the National Mental Health Policy Framework (2013–2020) acknowledges task-shifting as a critical approach to improving mental health nationally [44, 45]. Furthermore, BA is considered to be suitable for cross-cultural delivery because it targets behavioural change rather than beliefs and attitudes. To date, BA has not been tested amongst HIV-positive women in the perinatal period.

*The WHO/UNICEF CCD* package was developed to promote early childhood development, especially cognitive development, through responsive caregiving and early stimulation [46, 47]. Evaluations of CCD implementation have shown that the CCD strategy can be feasibly delivered by CHWs and there is good acceptability and demand from families [23, 48, 49]. Meta-analyses have shown significant benefits from programmes that support parents to enhance early stimulation as a means to improve early child cognitive development, including language development [50]. The CCD package has also been adapted and evaluated in several LMIC with positive results although it has not yet been tested in the context of perinatal depression or HIV, nor does it include content for pregnant women [50, 51]. Thus, the present trial is the first to adapt the CCD package for perinatally depressed, HIV-positive women. Furthermore, in this study, CCD has been augmented with specific content to begin during pregnancy including visual aids for lay counsellors, including information sheets and handouts.

### Objectives {7}

The primary objective is to test whether a home-based intervention, integrating behavioural activation for depression with a parenting programme adapted from CCD, for HIV-positive women with perinatal depression compared to Enhanced Standard of Care (ESoC) improves:

1. Maternal perinatal depression at 12 months postnatal
2. Child cognitive development at 24 months of age

#### Secondary objectives:

To identify if the combined intervention compared to the ESoC:

1. Improves maternal depression at end of pregnancy^1^ and 24 months postnatal.
2. Improves maternal anxiety at the end of pregnancy and 24 months postnatal.
3. Increases maternal adherence to Antiretroviral Treatment (measured as viral load (VL) and viral suppression post-initiation of treatment) over the trial period.
4. Increases rates of exclusive breastfeeding to six months postnatal.
5. Improves adherence to infant immunisation schedule over the 24-month postnatal period.
6. Reduces episodes of diarrhoea over the postnatal period^2^.
7. Improve the quality of infants’ cognitive and emotional stimulation within the home environment at 12 and 24 months.
8. Reduces child behavioural difficulties at 12 and 24 months postnatal.
9. Improves child language development at 24 months postnatal.
10. Improves child growth (infant height and weight) at 24 months postnatal.

### Trial design {8}

A cluster randomised controlled superiority trial with two parallel groups. The trial comprises 48–60 neighbourhood clusters, randomly allocated to the combined intervention or ESoC using a 1:1 allocation ratio with approximately 9–11 participants (mothers) per cluster, totalling 528 mother-infant pairs.

## Methods: participants, interventions and outcomes

### Study setting {9}

The trial is being conducted at the Africa Health Research Institute (AHRI) Somkhele Research Campus in rural Northern KwaZulu-Natal, South Africa, within AHRI’s Population Intervention Platform demographic surveillance area. The study area covers 845 km^2^ and the community is predominantly rural but contains an urban township and informal peri-urban settlements [53, 54]. The resident population is approximately 100,000 people (~ 20,000 households) of which the majority are isiZulu-speaking. The area includes one district-level hospital and 17 primary healthcare facilities. A 2019 study of a prospectively followed, population-based cohort from the study area estimated that HIV prevalence amongst women aged 15–54 years of age in the study area increased from 25 to 41% between 2005 and 2017 [55]. Despite high HIV prevalence, the incidence of HIV infection declined between 2012 and 2017 with men experiencing the biggest declines. Prevention of Mother-to-Child Transmission services were implemented in the sub-district in 2001 along with an HIV treatment programme in 2004 providing ART through public health facilities [56]. ART treatment is delivered in a decentralised model at primary health care clinics. Consistent with WHO guidance, all pregnant women not already on ART are initiated on ART treatment for life, irrespective of CD4+ count.

### Eligibility criteria {10}

**Fig. 1:**
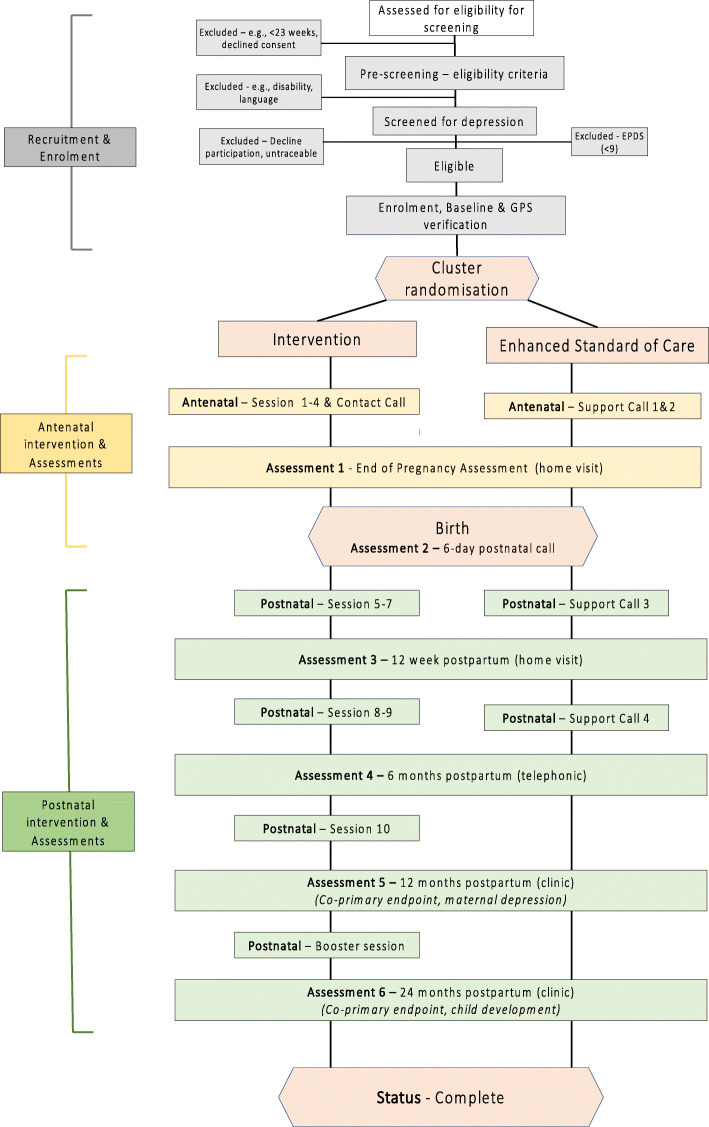
Participant flow

Participants provide written, informed consent both before screening and again if they fulfil all eligibility criteria before any trial activities, including randomisation, proceed. Minors (≥16 and < 18 years of age) provide guardian/parental consent as well as their assent to participate. The participant flow is outlined in Fig. 1.

#### Inclusion criteria:

- Pregnant women, 23–33 weeks of gestation at the time of enrolment;
- Participant willing and able to give informed consent for participation in the trial;
- Aged 16 years and above;
- Diagnosed HIV-positive;
- Participant meets the criteria for antenatal depression as defined by a score of ≥ 9 on the EPDS;
- Living, or planning to live, within the study area at the time of delivery and for at least 9 months after delivery (the intensive therapy period);
- Participant is conversant in isiZulu or English.

#### Exclusion criteria:

The participant may not enter the trial if any of the following apply:

- Any significant disease, disorder, or disability which, in the opinion of the Principal Investigator, may either put the participants at risk because of participation in the trial, or may influence the result of the trial, or the participant’s ability to participate in the trial. This includes hospitalisation for at least 3 days for severe psychiatric illness (specifically bipolar disorder, schizophrenia and any other psychoses), or a life-threatening or other serious physical illness (excluding HIV and tuberculosis).
- Current suicidal ideation/thoughts with specific plans and means identified.
- Substance or alcohol use disorder.
- Currently receiving psychological treatment for mental health problems.
- Participant planning to move away from the study area before 9 months postnatal.
- Participant not planning to cohabit with the infant.

### Who will take informed consent? {26a}

Screening consent is obtained before screening procedures. If potential participants are not able to be screened at the first meeting, initial consent to contact is obtained. The full informed consent is explained at the enrolment visit at the clinic and confirmed at the baseline assessment. For participants < 18 years, full guardian consent is obtained in addition to adolescent assent. Informed consent, confidentiality and data handling comply with Good Clinical Practice (GCP) regulations. The consent processes are conducted by trained recruiters who are supervised and monitored by a recruitment supervisor and the trial coordinator.

### Additional consent provisions for collection and use of participant data and biological specimens {26b}

No biological specimens will be collected in the trial.

## Interventions

### Explanation for the choice of comparators {6b}

Eligible participants are randomised in clusters to either the intervention or the Enhanced Standard of Care (ESoC) arms. The explanation for the choice of the intervention arm, which comprises a combination of behavioural activation (BA) and a parenting programme based on the WHO/UNICEF ‘Care for Child Development’ (CCD), is described in detail in the introduction (see ‘The Insika Yomama intervention’).

Enhanced Standard of Care (ESoC) was chosen as the control arm to provide telephonic counselling support and advice which also ensures that participants are helped to access care and referrals are made where necessary. The counselling support intervention includes four support and advice phone calls. The ESoC call programme was informed by the enhanced care package used in a recent perinatal depression trial in South Africa [57]. The standard of care in the study community is described in detail below.

### Intervention description {11a}

#### Therapy intervention

The intervention, integrating a psychological treatment for maternal depression and a parenting intervention to enhance early childhood development, is based on two evidence-based interventions delivered at the home:

1. Behavioural activation (BA) is a structured therapeutic approach that emphasises environmental causes of depression [37]. It is based on the evidence that increased activity (i.e. activation), and the resulting positive consequences, leads to a reduction of depressive symptoms. BA helps people understand the interaction between individual and environmental sources of their depression, and targets behaviours that might maintain or worsen the depression. Thus, BA aims to increase behaviours that are personally rewarding to improve mood and quality of life and decreases behaviours that maintain or worsen depression, such as passivity, avoidance and rumination. BA introduces small behavioural changes, building up the level of activity gradually towards long-term goals, making it feasible for perinatal women with limited time to spare.
2. WHO/UNICEF Care for Child Development (CCD) package is a parenting intervention that aims to enhance early childhood development, especially cognitive development, through improving parenting skills. The Insika Yomama parenting intervention tested in this study is adapted from the evidence-based CCD programme and aims to promote responsive caregiving and early stimulation. In this trial, we include specific pregnancy modules and visual aids to assist the delivery of the parenting content. The parenting component is designed to be integrated complementary to the BA component by ensuring that parenting activities enhance access to positive reinforcement through rewarding caregiving experiences, especially around responsiveness, thus potentially improving both mood and quality of parenting concurrently.

The participant is allocated a lay counsellor who provides all 10 sessions and the booster session.

##### Session structure

The combined intervention is delivered by lay counsellors in the participants’ homes across 10 sessions, starting in pregnancy (between 26 and  36 weeks of gestation) through to 9 months postnatal, along with an additional booster session at 16 months postnatal. The initial session lasts up to 2 h and focuses on orientation to BA and assessment of behaviours around depression using a BA diagram to conceptualise problem behaviours. The session focuses on BA only. The remaining sessions last approximately 1.5 h each, compromising combined mother-focused BA modules and infant-focused parenting components. The therapy arm also receives a ‘keep in contact call’ at 36–38 weeks of gestation between therapy session 4 and delivery.

##### Materials

Electronic tablets assist the lay counsellors in delivering the combined intervention and keeping track of participants’ developments and session content. The tablets contain visual aids and a treatment manual. The treatment manual has been developed to guide the lay counsellors in providing standardised BA activities. This is accompanied by handouts containing exercises and health information relating to antenatal care, breastfeeding, management of infant crying and HIV treatment are provided for participants to accompany the sessions.

The BA comprises:

- Assessment—Session 1 is an orientation to BA (explanation of core concepts); psychoeducation around self-care, routines, and nourishing activities (i.e. activities one enjoys doing); and setting of treatment goals.
- Activating activities—Sessions 2–6 cover the core BA content. The treatment manual includes modules on self-care (sleep, eating, exercise), adherence to medication, routines, nourishing activities (e.g. bath with privacy) and problem-solving. Each module focuses on behavioural change that increases positive reinforcement and reduces avoidance behaviour. By the end of each session, homework is discussed (e.g. activation goals, mood monitoring and avoidance behaviours in the period leading up to the next session).
- Planning for the future—Session 7 involves identifying strategies that have been helpful and setting goals for the future.
- Review and consolidation sessions—Sessions 8–10 review progress, reinforce maintenance of changes and set further goals.
- The core principles of BA are reviewed during the booster session (16 months postnatal) and any new difficulties that arise are dealt with using skills developed in the earlier sessions.

The Parenting Intervention components focus on:

- Increasing attention to infant facial and verbal cues.
- Increasing ‘contingent responding’, by guiding the participant to attend to her baby’s signals and efforts at communication, and to respond to her baby’s communication in a way that is synchronous with the baby’s signals and focus.
- Increasing opportunities for early stimulation.

The behaviour change techniques employed include:

- Providing opportunities for participants to try age-appropriate play interactions with their infants and receive coaching and feedback on ways to enhance the interaction.
- Using visual aids such as homemade/low-cost toys, illustrated case studies and prompts.
- Problem-solving with participants about ways to overcome barriers to providing early stimulation and nurturing care.

**Fig. 2:**
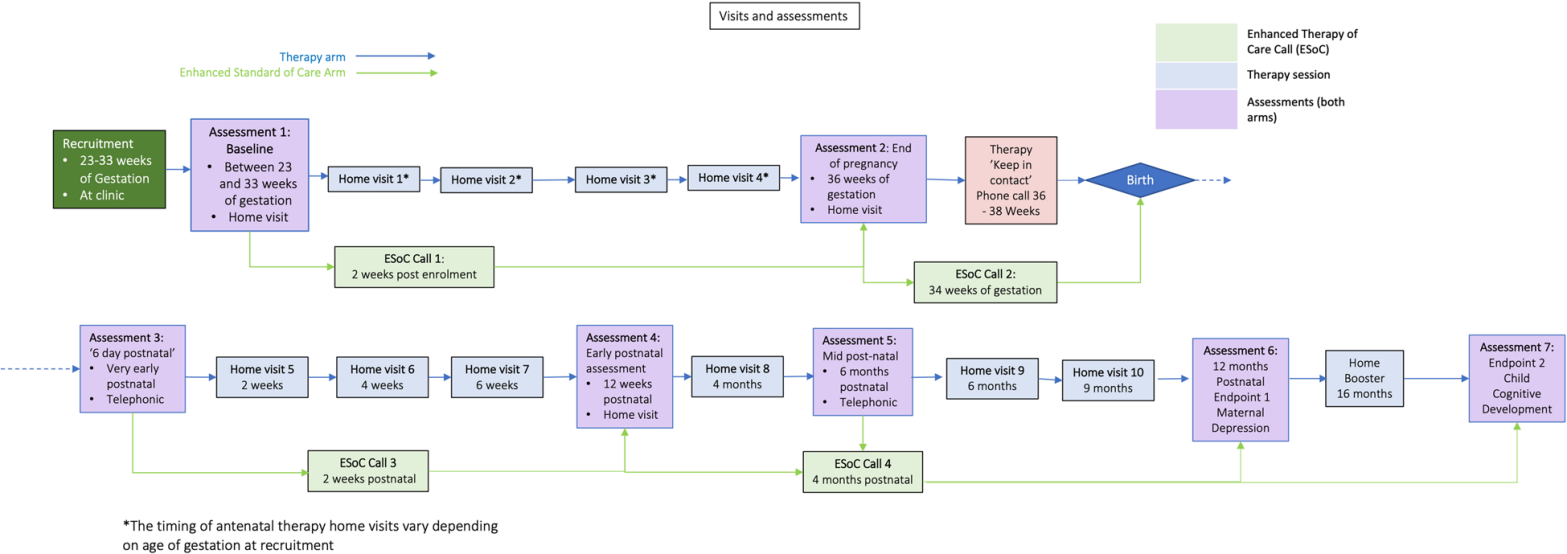
Participant timeline

The session content is distributed as follows (see Fig. 2):

Antenatal (sessions 1–4)—Core parenting principles and activities are introduced through audio-visual material, including demonstration videos, to prepare the participant for the baby. Activities include attending to and recognising infant facial and vocal expressions, contingent responsiveness and communication through singing.

Postnatal (sessions 5–10)**—**Extends the core principles and activities introduced antenatally and supports the participants in applying these during interactions with their baby. Additionally, new modules are introduced, including guidance around breastfeeding (including an emphasis on EBF); different types of play (developmentally appropriate activities, e.g. simple face-to-face games and ‘peek-a-boo’) with emphasis on contingent responding; and supporting the participants in consulting their Road to Health Book to recognise signs of serious illness in the infant.

These sessions are delivered from 2 weeks postnatal until 9 months postnatal.

In the booster session, age-appropriate opportunities for a range of play activities are provided, because of the rapid advances in development in the second year. The core principles of the parenting intervention are also reviewed and any new difficulties that arise are dealt with using skills developed in the earlier sessions.

##### Personnel—recruitment, training, and supervision

The intervention is delivered by experienced lay counsellors who have at least 2 years of counselling experience, including working with women and children and HIV counselling. Intervention counsellors receive 6 weeks of training based on the treatment manual, followed by a period of supervised practice. No counselling sessions are attempted by the counsellors until they have been assessed as competent by the supervising psychologist (using standardised checklists and through observation of role-playing). The lay counsellors’ first sessions are reviewed, and any competency issues are addressed through additional training. The counsellors receive weekly supervision from the trial psychologist as well as added supervision from therapy supervisors who are more senior counsellors. Ongoing training is also conducted through workshops.

##### Fidelity

The fidelity and competency of the lay counsellors are monitored through several measures:

- The lay counsellors complete a Fidelity Checklist for every module.
- Sessions are audio-recorded (if participants consent) and a sample of audio recordings is scored by the supervising psychologist and feedback is provided as needed.
- The trial psychologist conducts periodic in-person therapy observation with all therapists to identify any additional training needs.
- A sample of audio recordings is scored by an independent assessor to provide a formal assessment of fidelity to the intervention.
- Weekly supervision meetings are conducted by an experienced psychologist who records fidelity and competence. Retraining is conducted as needed.

#### Enhanced Standard of Care (ESoC)

##### Standard of Care in the community

In the study community, mental health services for all women in the perinatal period are coordinated from the local Department of Health (DoH) district hospital. The local DoH district hospital includes a psychology department, with a full-time clinical psychologist and psychiatric nursing staff as well as a crisis centre. The crisis centre provides counselling and forensic services to victims of gender-based violence, delivered by the psychologist, district surgeon, and medical and nursing staff. The local DoH district hospital provides acute care for psychological and psychiatric emergencies, whilst patients requiring higher levels of care are referred to tertiary centres. The psychology department provides inpatient and outpatient care. Furthermore, the staff from the psychology department rotate to the local primary health clinics. There is a psychiatric unit (inpatient and outpatient facilities) in a tertiary referral hospital in a neighbouring district. A psychiatrist from the unit provides remote consultation to staff at the local district DoH hospital as well as holding monthly ward rounds and a psychiatric outpatient clinic at the hospital.

In addition to this standard of care available to women in the perinatal period in the study community, the study team provides training in the identification, initial management and referral of perinatal mental health problems to all antenatal care staff at the recruiting clinics. The training is informed by materials and support provided by the Perinatal Mental Health Project, based at the University of Cape Town [58] , and delivered by an experienced clinical psychologist. To ensure standardisation across the clinics, the training was conducted at the beginning of the trial and is refreshed periodically every 6 months throughout the trial. Women in both arms of the trial have access to these primary health care and hospital-based mental health services.

##### Enhanced Standard of Care (ESoC)

In addition to the standard of care in the district, the ESoC arm receives a telephonic counselling support and advice intervention which also ensures referrals and access to care where necessary. The counselling support intervention includes four support and advice phone calls. The ESoC call programme was informed by the enhanced care package used in a recent perinatal depression trial in South Africa [57].

The telephone counselling uses questions, guided by standardised scripts, and supportive listening to enable the caller to assess the participant’s current emotional wellbeing and health, and to ensure referrals and access to care where necessary. The caller (who has had at least 2 years of counselling experience) provides advice and supports problem-solving around managing their mental and physical health as well as partner and family relationships. Scripts for each call have been developed to support the caller in dealing with these issues. The caller also guides participants to use the services and support provided by the district DoH services. Training and supervision support the ESoC caller to be empathic and responsive to the participants. The caller also identifies risk situations, including suicidal ideation, that have arisen. The caller reports and refers these to the trial risk management team in line with the trial risk management protocol for further action. ESoC participants receive all four support calls from the same support caller. The participants are also provided with a study telephone contact number in case of urgent need and are given telephone credit.

In addition to the phone calls provided to the ESoC arm, participants are assessed on 7 occasions as part of the trial (baseline and six follow-up assessments) (both arms) by trained assessors which enables the identification of problems of significant clinical risk or concern. When these arise, they are evaluated by the team which includes a full-time on-site clinical psychologist. This is followed by initial management and onward referral to medical, psychological, and social services depending on the case. Psychological cases are managed by the trial risk management team and the AHRI Clinical Research Department. An initial assessment is always provided by the trial clinical psychologist. Where appropriate, the psychologist undertakes initial management and follow-up to ensure timely assessment and care without overburdening the local DoH services. The trial psychologist liaises with and makes referrals to the local DoH psychologist and the Department of Social Development (DSD) (social services) as necessary. Support for medical and social cases is provided by the AHRI Clinical Research Department. In collaboration with this department, the trial has established referral pathways and working relationships to the local DoH and DSD. In addition, all referrals are actively monitored until care has been accessed and, where necessary, the trial team continues to follow up.

##### Session structure of ESoC calls

Participants receive two antenatal calls (2 weeks post-enrolment and at 36 weeks of gestation) and two postnatally (2 weeks and 4 months postnatal). Each call follows the same structure:

1. Connect and check-in—To enquire about their emotional, social and physical wellbeing.
2. Suggestions and advice—Based on the participant’s responses, the ESoC caller uses scripts to offer suggestions and advice on actions the participant can take to manage their mental health, health or relationship difficulties.
3. Health information—The ESoC caller offers health information messages linked to the participants’ stage of pregnancy or parenting and offers advice on the availability of services and makes referrals where needed.

In addition, brief health information is provided to the participant during each call:

1. Early Pregnancy Call—Information on attending antenatal visits, managing HIV treatment;
2. Late Pregnancy Call—Information on managing HIV treatment, healthy pregnancy and planning for delivery;
3. Early Postnatal Call—Information on infant feeding, managing infant’s sleep and routines;
4. Late Postnatal Call—Information on infant feeding, managing infant’s sleep and routines, managing HIV treatment, parenting

##### Materials

Electronic tablets assist the ESoC caller with scripts to deliver the ESoC calls and to keep a record of session content. The caller also completes a checklist for each call.

A parenting leaflet developed by UNICEF South Africa (with the Department of Education) is given to all participants in the study at the clinic at enrolment.

##### Personnel—recruitment, training, and supervision

The ESoC is delivered by an experienced ESoC caller who has at least 2 years of counselling experience, including working with women and children, and providing HIV counselling. The ESoC caller attends a 4-week intensive training workshop followed by a mock telephone call competency assessment and intensive supervision over the first 2 months. Periodic refresher training is provided. The training includes the identification of risk situations, including suicidal ideation. The ESoC caller receives weekly supervision and debriefing with a trained counselling supervisor, to support and facilitate referrals in risk cases and in managing the logistical aspects of the ESoC. The caller contacts the ESoC supervisor between supervision sessions if issues of concern or uncertainty arise.

##### Fidelity

The fidelity and competency of the ESoC callers are monitored through several measures including supervision, debriefing and recordings and rating of ESoC calls (if participants consent).

### Criteria for discontinuing or modifying allocated interventions {11b}

Criteria for discontinuation of allocated interventions include situations where participants have requested discontinuation or have or have developed another major physical illness or injury which makes it too challenging for the participant to continue. In specific situations, such as following bereavement or a major life event, the Principal Investigators can agree to a limited number of additional therapy sessions or ESoC calls.

### Strategies to improve adherence to interventions {11c}

Several strategies are implemented to improve adherence, including clear communication to the participants about intervention timing and structure (including the provision of an intervention schedule and homework sheets). Text reminders and/or calls to participants are made when therapy/ESoC sessions are missed. Data is routinely collected during each intervention session using checklists to monitor adherence to intervention content which are frequently reviewed. 

### Relevant concomitant care permitted or prohibited during the trial {11d}

Concomitant psychopharmacological treatment is not commonly prescribed in the population under study. For both the intervention and ESoC groups, where it is deemed necessary by a health care provider, concomitant psychopharmacological care is permitted, and carefully documented and monitored. Additional care in the form of psychological therapy or treatment is permitted in circumstances to manage risks such as significant suicidality or significant social harm, such as domestic violence. In such cases, we make appropriate referrals to health or social services following well-established referral pathways and risk management protocols. Furthermore, for the ESoC arm additional referrals and interventions for psychological or social care in the public services are supported and encouraged as part of the four support and advice calls. Careful records are kept of all referrals. Provision and participation in supplementary child stimulation activities, and/or attendance at childcare or educational centres or nursery schools is permitted across both allocations.

#### Provisions for post-trial care {30}

A trial clinical psychologist is employed on a full-time basis to supervise intervention delivery and to provide ancillary psychological care to participants in emergencies who meet criteria, including those expressing suicidal ideation and intent, those experiencing life-threatening trauma, violence and/or close family bereavement. In cases of infant death or stillbirth, additional bereavement sessions are offered. Following the final trial outcome assessment (24 months postpartum), all participants with high-risk profiles will be assessed by the trial psychologist, who will provide initial management. Those participants who require additional and ongoing care will be referred to the local DoH and DSD services as necessary. Additionally, psychological sessions may also be offered to participants by the trial psychologist. If the therapy intervention is shown to be successful, in line with the pre-trial consultations, we will train local primary healthcare workers to provide the combined therapy locally.

#### Outcomes {12}

##### Primary outcomes

The following primary outcomes are shown in Table 1:

1. Child cognitive development at 24 months of age, assessed using the Bayley Scales of Infant and Toddler Development III (BSID-III) cognitive subscale.
2. Maternal perinatal depression at 12 months postnatal assessed using the Edinburgh Postnatal Depression Scale (EPDS).

**Table 1:** Primary outcomes

Domain	Measure	Metric	Method of aggregation	Timepoint
Child cognitive development at 24 months of age	Bayley Scales of Infant and Toddler Development III (BSID-III) cognitive subscale (Composite Score)	Mean difference	Mean and SD	24 months postnatal
Maternal depression	Edinburgh Postnatal Depression Scale (EPDS), total score	Mean difference	Mean and SD	12 months postnatal

##### Secondary outcomes

The 10 secondary outcomes and the associated measures are summarised in Table 2 and Table 3.

**Table 2:** Secondary outcomes

	Domain	Measure	Metric	Method of aggregation	Timepoint
1	Maternal depression	Edinburgh Postnatal Depression Scale (EPDS), total score	Mean difference	Mean and SD	End of pregnancy; 24 months postnatal
2	Maternal anxiety	Generalized Anxiety Disorder 7-item (GAD-7) scale, total score	Mean difference	Mean and SD	End of pregnancy, 24 months postnatal
3	Maternal antiretroviral treatment adherence	Viral Suppression (Yes/No)	Risk ratio	Frequency and percentage	End of pregnancy; 12 weeks, 12 months, 24 months postnatal
4	Exclusive breastfeeding	Exclusive breastfeeding at 6 months (Yes/No)	Risk ratio	Frequency and percentage	6 months postnatal
5	Adherence to infant immunisation schedule over the 24-month postnatal period	No. of immunisations recorded	Mean difference	Mean and SD	12 weeks, 12 months, 24 months postnatal
6	Infant diarrhoea	Any maternal report of diarrhoea over the previous 14 days.	Risk ratio	Frequency and percentage	12 weeks, 6 months, 12 months, 24 months postnatal
7	Cognitive and emotional stimulation within the home environment	Early Childhood Development section of Multiple Indicator Cluster Surveys (MICS6) Questionnaire for cognitive and emotional stimulation for children under five, total score	Mean difference	Mean and SD	12 months, 24 months postnatal
8	Infant behaviour	Parenting Stress Index, Short Form (PSI/SF: parent-child dysfunctional interaction subscale and difficult child subscale, total score	Mean difference	Mean and SD	12 months postnatal
		Externalising subscale of Child Behaviour Checklist (CBCL), total score	Mean difference	Mean and SD	24 months postnatal
9	Child language development	Language subscale of the Bayley Scales of Infant and Toddler Development III (BSID-III), Composite score	Mean difference	Mean and SD	24 months postnatal
10	Child growth (infant height and weight at 24 months)	Road to Health Book, study Weight and height measurements	Mean difference	Mean and SD	24 months postnatal

**Table 3:** Mediators, including measures and timepoints

Mediators	Measure	
Family, relationship support and conflict	Relationship conflict questions adapted from the Romantic Partner Conflict Scale [59]. Support questions used from social support measure previously validated in South Africa [60].	Baseline, end of pregnancy, 12 weeks, 12 and 24 months
Rumination	Brief Rumination Response Scale [61]	Baseline, end of pregnancy, 12 weeks, 12 and 24 months
Maternal recognition of infant faces and sounds	Stimuli task	Baseline, 12 months

#### Participant timeline {13}

Participant timeline is shown in Table 4.

**Table 4:** Participant timeline

Assessment (A)		A1	Allocation	A2	Birth	A3	A4	A5	A6	A7
	Enrolment and screening			Post-allocation period						
Timepoint	Screening and Enrolment	Baseline		End of pregnancy		6–12 days	12 weeks	6 months	12 months	24 m
Screening form and eligibility checklist	X									
Demographic and socio-economic status		X								
Tracing and location information	X	X		X			X	X	X	X
GPS capture of home		X								
Allocation			x							
**Primary outcomes**										
BSID-III cognitive										X
EPDS									X	
**Secondary outcomes**										
EPDS	X			X			X			X
GAD-7		X		X					X	X
Maternal Antiretroviral treatment adherence		X		X			X		X	X
Exclusive Breastfeeding								X		
Infant immunisations							X		X	X
Infant diarrhoea							X	X	X	X
Cognitive and emotional stimulation (MICS)									X	X
Infant Behaviour (PSI/SF and Externalising subscale CBC)*									X	X
BSID-III language										X
Child Growth (weight and height)							X		X	X

#### Sample size {14}

The first primary outcome is the cognitive subscale on the BSID-III at 24 months of age. To achieve power of over 90% (two-sided *t*-test with a significance level of 0.05), and assuming an estimated difference of 6 points (SD 15), a total sample size of 396 women (198 per arm) is required. This calculation takes into account geospatial clustering (28 clusters per arm with an intra-cluster correlation coefficient (ICC) of 0.05) and ‘counsellor effect’ in the intervention arm (4 lay counsellors with an ICC of 0.05). To take account of attrition of up to 25%, a total sample size of 528 women is being recruited (264 per arm, 48–60 geospatial clusters, 9–11 women per cluster).

Using the EPDS assessment with a standard deviation of 5, with the same assumptions of clustering as above, a difference of 2 points between trial arms (not adjusting for baseline or repeated measurements) could be detected with 90% power and a 5% two-sided significance level. Analysis using repeated measures, taking into account within-participant correlation over time, would allow smaller differences to be detected with the same power

#### Recruitment {15}

Recruitment and screening take place from 15 DoH Primary Health Care Clinics within a sub-district in KwaZulu-Natal offering maternity services.

##### Recruitment strategy

The recruitment process is led by recruiters from the trial team with support from DoH antenatal nurses and clinic clerks.

Recruitment into the trial occurs through a four-stage process:

1. Identification of potential participants—Clinic records are reviewed by antenatal nurses and clinic clerks at antenatal clinics to identify HIV-positive, pregnant women (gestational age 23–33 weeks) over the age of 16 years. The nurses and clerks reassure the potential participants that their clinical care will not be compromised by participation.
2. Referral to recruiter—If the identified potential participants consent, they are referred to a trial recruiter who explains the trial procedures and screens for additional eligibility criteria.
3. Depression screen—Administration of the EPDS if the potential participant meets all other eligibility criteria.
4. Consent processes—Written consent and scheduling of baseline assessment at home for participants who fulfil all inclusion criteria, including an EPDS ≥ 9.

The baseline assessment is conducted at the home at which point the GPS location is verified. The participant is randomised (based on their geospatial cluster) following the baseline assessment.

Only participants who were diagnosed with HIV at least 2 weeks previously and have been initiated on ART are recruited.

If during the screening or recruitment process a participant indicates thoughts of self-harm on the self-harm item of the EPDS, this initiates a risk management response and immediate notification is then made to the Trial Coordinator and Trial Psychologist. The Trial Psychologist then makes a psychological assessment in order to establish whether there is active suicidal intent or behaviours. It these are present, the psychologist undertakes initial management including telephonic follow-up at 24 h, 48 h and 72 h, and referral to the DoH Psychologist at the local hospital as appropriate. Following this management, if the potential participant is no longer at risk for self-harm, and still meets recruitment criteria, then they will be offered participation in the study.

### Assignment of interventions: allocation

#### Sequence generation {16a}

The unit of randomisation is the cluster using the geospatial location of the participant’s home. There are approximately 300 neighbourhoods in the region included in this trial; these are defined by geographical area as well as population density so that they are equivalent in terms of sample size. The distinct neighbourhoods have been merged into 48–60 clusters to ensure comparable clusters in terms of key indicators, including population size. This clustering approach, and the important role of randomisation in this trial, has been presented to, and approved by, the AHRI Community Advisory Board.

#### Concealment mechanism {16b}

Allocation concealment is ensured by a two-step enrolment procedure whereby neither the recruiter nor the assessor establishing eligibility know to which arm the clusters have been allocated.

#### Implementation {16c}

The 48–60 geospatial clusters are randomly allocated to the integrated intervention or ESoC with an allocation ratio of 1:1 using a random sequence generated by a senior statistician at the National Perinatal Epidemiology Unit (NPEU), University of Oxford (using Stata/SE version 13 for Windows). The randomisation schedule is sent to AHRI using a secure web link and implemented by the local data management team. None of these parties is involved in the implementation of trial activities (recruitment, assessments, therapy).

### Assignment of interventions: blinding

#### Who will be blinded {17a}

Participants are not informed at enrolment as to which arm they are allocated to ensure the blindness of the recruiters and assessors. Rather, participants are informed that a trial counsellor will contact them. The intervention counsellors or the ESoC caller reveal the arm to which the participant has been allocated when they first make contact.

The recruiters and assessors are blinded to the treatment allocation arm. Furthermore, the assessors are independent of the lay counsellors performing the counselling sessions and blind to treatment allocation. The two primary endpoints are assessed by different independent assessors (at 12 and 24 months).

#### Procedure for unblinding if needed {17b}

In the event of inadvertent unblinding of an assessor, standard operating procedures are in place to reduce the impact and ensure that the assessor is never allocated to the given participant in future assessments.

##### Minimisation of contamination

Recruitment staff are located at clinics whilst intervention staff operate from a separate AHRI campus to minimise contact between recruitment and other staff. ESoC caller and lay counsellors operate separately within different scopes of work in distinct offices, are line-managed and supervised by different individuals, use separate transport services and attend separate meetings to reduce the risk of unblinding. They are trained not to share information that could compromise the blinding or the integrity of the trial. Any instances of unblinding are recorded and assessed at the conclusion of the trial.

### Data collection and management

#### Plans for assessment and collection of outcomes {18a}

Participants in both arms receive 6 assessments following the baseline assessment: (1) end of pregnancy; (2) 6-day postnatal screener, (3) 12-week postnatal, (4) 6 months; (5) 12 months, (6) 24 months. The points at which primary and secondary outcome data are collected are outlined in Tables 1, 2, and 3. Assessments take place at home, except for the 24 month assessment, including the primary endpoint BSID-III assessment, which takes place in participating clinics.

Table 5 outlines the standardised data collection tools used in the study. Below we outline tools specific to this study.

**Table 5:** Data collection tools

Instrument	Description	Outcome	Contextual validity
The Bayley Scales of Infant Development-Third Edition (BSID-III) [62]	A comprehensive objective assessment administered face-to-face by a qualified independent assessor to assess child development. Only the cognition and language subscales are administered.	Child cognitive and language development	Validated in South Africa [63, 64] with reported values similar to the reference population in the USA [62].
Edinburgh Postnatal Depression Scale (EPDS) [65]	10-item questionnaire of perinatal depressive symptoms over the last 7 days assessed on a scale of 0 to 3.	Maternal depressive symptoms	Widely used and validated in Africa and South Africa, including use amongst antenatal populations [66]. Has been validated in the study population against a structured clinical interview for depression (DSM-IV) showing good specificity (93%) and sensitivity (68%) for detecting clinical depression [67, 68]
Child Behaviour Checklist (1.5-5-year-old) [69]	Externalising subscale (attention problems and aggressive behaviour syndrome scales) of the CBCL. 24 items, assessed on a scale of 0 to 2. Parental self-report.	Child externalising behaviour	Has been shown to be reliable in Africa [70], and to have high (> 90%) sensitivity and specificity for identifying behavioural emotional problems compared to a clinical diagnosis by a psychiatrist in other LMICs [71] Widely used and well-validated in South Africa [72, 73].
Parenting Stress Index Short Form [74, 75]	Measure of parenting stress related to three domains: the parental role, the parent-child relationship, and the degree to which the parent finds the child difficult. The scale comprises 36 statements, which are scored 1 (strongly disagree) to 5 (strongly agree) and can be summed to reflect the total score for each domain.	Maternal perception of child behaviour (Dysfunctional Interaction and Difficult Child subscales)	Has been shown to be reliable in South Africa [76]
Brief Rumination Response Scale	A 5-item questionnaire assessing depressive rumination assessed on a scale of 0 (never) to 4 (always).	Maternal rumination	A validated and reliable measure of depressive rumination [61, 77]
The Generalized Anxiety Disorder Scale (GAD-7)	7-item questionnaire assessing symptoms of generalised anxiety disorder over the previous 2 weeks. Scores	Maternal anxiety	Has been widely used in the study setting and has shown good reliability [76, 78].
Multiple Indicator Cluster Survey (MICS)	Specific questions from the Early Child Development module of the UNICEF ‘Multiple Indicator Cluster Surveys’ (MICS) under five are used to assess cognitive and emotional stimulation at home. Items are mostly scored on a dichotomous scale of 0 and 1 except for the question about how many children’s books or picture books a mother has for her child (0 = none, 1 = a number of books and 2 = ten or more books).	Cognitive and emotional stimulation.	Validated survey used in over 100 countries over the past two decades, also used in large scale surveys in 27 African countries [79, 80].

### Maternal recognition of infant faces and sounds

The participant’s ability to accurately interpret and respond to infants’ emotional cues is assessed using a subset of stimuli baby faces developed by rigorous validation methods in Brazil [81] and a sample of standardised baby vocalisations [82, 83]. Mothers are asked to indicate their interpretation of both the facial expressions (‘happy’, ‘neutral’, ‘sad’) and the vocalisations (positive ‘laughs’ neutral ‘babbles’ and ‘negative ‘cries) on an electronic tablet screen using a vertical visual analogue scale. The faces used for this sample include Caucasian, African and mixed-race infant faces and the images are presented in greyscale and matched in size and luminosity [84]. The task takes around 15 min to complete.

### Economic evaluation

Costing is conducted from a societal perspective, including financial costs to providers, opportunity costs of diverted provider resources (such as time of staff not paid on the trial, space) and financial (e.g. travel, time off work) and opportunity costs (e.g. time away from unpaid productive activities) to participants. We distinguish between research costs and operational costs to allow estimation of the cost of replication in non-research settings. The analysis involves three levels with increasing complexity. The first level involves a cost-utility analysis of the intervention with the primary and secondary outcome included in turn: the cost per improvement in cognitive development; the cost per improvement in maternal depression. The second level involves cost-effectiveness analysis using disability-adjusted life years (DALYs) averted. Improvements in child cognitive development and maternal depression are converted to DALYs based on assumptions regarding the duration of the observed benefit. The cost per DALY is then calculated. The third level compares costs to a vector of benefits, including indicative estimates of long-term gains (based on simple models), such as improved education outcomes and increased income in adulthood.

### Nested qualitative study

A qualitative sub-study is conducted alongside the trial, using semi-structured interviews with both the lay counsellors who delivered the intervention and with a sample of participants from each study arm. The sub-study aims to investigate the acceptability, challenges, enablers and potential benefits of the combined intervention compared to ESoC. The sub-study also investigates the acceptability and effectiveness of the therapist training and therapy supervision for this intervention. The data will be analysed using grounded theory principles and thematic content analysis [85, 86].

### Plans to promote participant retention and complete follow-up {18b}

We have developed a standard operating procedure to guide and promote retention and follow-up. This includes maintaining a regular schedule of assessments. Tracing and follow-up methods include text, telephone calls and in-person track and tracing. Participants are encouraged to notify the trial staff if they change their telephone number or address. Retention in the study community in previous clinical trials using these strategies has resulted in high retention rates. If participants discontinue the intervention (therapy/ESoC), they continue to receive assessments, including the primary outcome assessments (providing their consent).

### Data management {19}

Study data are collected and managed using a secure, web-based data collection platform on encrypted tablet computers which are securely uploaded to a central server hosted at AHRI. The platform is a secure, web-based software platform designed to support data capture for research studies, providing (1) an intuitive interface for validated data capture; (2) audit trials for tracking data manipulation and export procedures; (3) automated export procedures for seamless data downloads to common statistical packages and (4) procedures for data integration and interoperability with external sources. The tablets are encrypted, and all documents are stored safely under confidential conditions. On all trial-specific documents, other than the signed consent forms, the participant is referred to by the trial participant number, not by name.

### Confidentiality {27}

The trial staff ensure that the participants’ anonymity is protected as far as possible. The trial complies with GCP and established practice which requires data to be anonymised as soon as it is practical to do so. Audio and video recordings are stored on a secure server with very limited access (to senior research team members and researchers working directly with a particular participant or directly involved in coding data). Participants are informed that audio-visual recordings will not be used outside of the team of researchers, and this is outlined in the consent form. Following the completion of the trial, the data will be downloaded and de-identified.

### Plans for collection, laboratory evaluation and storage of biological specimens for genetic or molecular analysis in this trial/future use {33}

Not applicable, no biological samples are collected.

### Statistical methods

#### Statistical methods for primary and secondary outcomes {20a}

The primary inference will be based on the BSID-III cognitive score at 24 months and the EPDS at 12 months. For the primary outcomes and other continuous outcomes, the mean (SD) will be presented by the allocation group, and the mean difference (plus 95% confidence interval) will be estimated using mixed-effects linear regression. For binary outcomes, the number and percentage with the outcome will be presented by the allocation group, and the risk ratio (plus 95% confidence interval) will be estimated using a mixed-effect binomial or Poisson regression model.

The unit of randomisation is a geospatial cluster as outcomes are collected at the individual level, hence the unit of analysis is the mother and the infant. There is also an additional level of clustering by the counsellor delivering the intervention. The lack of independence amongst individuals in the same cluster and standard methods of analysis will underestimate the standard error of the treatment difference yielding *p* values that are too small. To account for the correlation of outcomes within clusters, the geospatial cluster and counsellor identifier will be fitted as random effects, with counsellor nested within the geospatial cluster. The intra-class correlation coefficients for geospatial cluster and counsellor will be estimated.

For the EPDS and the GAD-7, a repeated measures model will be fitted, including the baseline, end of pregnancy and 12-month and 24-month scores. For the cognitive and emotional stimulation at home outcomes, a repeated measures model will be fitted including the 12- and 24-month scores. Mixed-effects models with maximum-likelihood estimation allow participants with incomplete repeated measures data to be included in the model, contributing to the estimation of model parameters. The mean scores with 95% confidence intervals will be plotted over time by the allocation group.

In addition to adjusting for important baseline differences between randomised groups, any differences between participants followed up to 24 months and those lost to follow-up will be adjusted for in the final models. Both unadjusted and adjusted models will be fitted, but the primary inference will be based on the adjusted model which provides unbiased estimates if the outcome data is missing at random (i.e. only dependent on observed characteristics).

#### Interim analyses {21b}

A Data Monitoring and Safety Board (DSMB), independent of the trial organisers and sponsors, has been established with the remit to review trial progress. The terms of reference for the DMSB were agreed upon at their first meeting and documented in the DSMB charter. The DSMB is chaired by a senior clinical trialist, and members include two statisticians and a clinician. Interim data analyses are supplied, in strict confidence, to the DSMB, as frequently as the chair requests and meetings are held at least annually. Based on interim data on the trial’s outcomes, adverse event data, accumulating evidence from other trials and any other relevant evidence, the DSMB will inform the Trial Steering Committee (TSC) if, in their view, there is proof beyond a reasonable doubt that the data indicate that any part of the protocol under investigation is either clearly indicated or contra-indicated, either for all trial participants or for a particular sub-group of trial participants. A difference of at least 3 standard errors in the interim analysis of a major endpoint may be needed to justify halting, or modifying, such a study prematurely.

#### Methods for additional analyses (e.g. sub-group analyses) {20b}

Pre-specified sub-group analysis of factors known to be associated with infant cognitive development—maternal education, socio-economic class, the severity of depression at trial entry and infant sex—will be performed for the infant cognitive development outcome at 24 months using the statistical test of interaction. Sub-group analysis based on maternal education, socio-economic class and infant sex concerning maternal depression at 12 months will also be performed. The sub-groups will be categorised as follows:

- Maternal education (primary completed or below; grade 10; matriculation or above).
- Socio-economic status (paid employment yes/no).
- Severity of depression at trial entry (continuous).
- Infant sex (male/female).

#### Methods in analysis to handle protocol non-adherence and any statistical methods to handle missing data {20c}

Women with outcome data will be analysed in the groups to which they are randomly assigned, regardless of deviation from the protocol or treatment received (modified ITT population). Women whose infant died during the trial will also be included unless they withdrew consent to participate further in the trial.

The number and percentage of individuals missing data for outcome measurements that are based on summing items to give overall scores (e.g. EPDS, GAD-7, and subscales of the PSI/SF and CBCL) will be described. Missing items will be imputed if ≤ 20% are missing using the median score of completed scale items unless alternative guidance is provided in the scoring manual.

A multiple imputation analysis will be performed for the primary infant and maternal outcome if attrition exceeds 5%. The multiple imputation model will include baseline characteristics and outcome measures collected before the missing assessment, which are associated with missing status.

#### Plans to give access to the full protocol, participant level-data and statistical code {31c}

Data will be available beginning 9 months and ending 36 months following main article publication to researchers who provide a methodologically sound proposal that purposes to achieve aims in the approved proposal and /or for individual participant data meta-analysis. Data is documented and stored on the AHRI Data Repository (https://data.ahri.org) with a digital object identifier (doi) and can be accessed with permission and in line with AHRI policies and procedures. Data requestors will need to sign a data access agreement before any data can be shared. In addition, Study Protocol and Statistical Analysis Plan documents will be available. Data sets associated with publications will be made available in line with Wellcome Trust data policies and journal requirements.

### Oversight and monitoring

#### Composition of the coordinating centre and trial steering committee {5d}

This trial is managed and coordinated by a trial group (management, operational and data management sub-groups) with regular advice and consultation from the investigators’ group and the AHRI Clinical Research Department.

The trial group composes of management, operational and data management sub-groups. The management sub-group consists of the principal investigators, trial manager, trial coordinator, and trial psychologist. The group meets at least once a week virtually to discuss and manage major trial operational challenges (screening, recruitment, assessments, therapy/ESoC), logistics, adverse events (AE) and severe adverse events (SAEs), staffing, and trial reporting and monitoring. Trial reporting and monitoring includes the management and reporting of AEs and SAEs and trial progress using summary statistics and graphs**.** The operational sub-group includes the four trial components (recruitment, assessment, therapy, ESoC) and is managed by the trial coordinator. The operational teams meet at least once a week, with individual meetings and contact points as needed. The four teams are operated separately to ensure blinding. The data management subgroup meets weekly and includes the AHRI data manager, trial manager and trial coordinator.

The investigators’ group consist of the trial management group and experts (investigators) in maternal health and early child development. The investigators are consulted regularly dependent on their specific roles and small sub-group meetings are organised as needs be. The whole group meets formally every 3 months. Trial Progress Reports containing updates, summary statistics and graphs and discussion points are submitted for these meetings.

The trial group confers regularly with the AHRI Clinical Research Department, primarily regarding SAE and AE management. The trial coordinator has frequent meetings with the head of the Clinical Research Department and attends regular AHRI wide project coordination meetings to ensure that the trial implementation is coordinated with awareness of other research activities in the study community.

The Trial Steering Committee (TSC) acts as the oversight body for this trial on behalf of the Sponsor/Funder and it should also provide advice through its independent Chair on all aspects of the trial. All substantial issues regarding the trial must go to the TSC for consideration. The Data Safety Monitoring (DSMB) is advisory to the TSC and the DSMB makes recommendations to the TSC based on the interim data. The TSC oversees the timely analysis, writing up and publication of the main trial results. The independent members of the TSC will have the opportunity to read and comment on the proposed main publications of trial data before submission. The TSC is made up of the chair, three independent members, including an experienced trials statistician. The TSC meets with the trial management team and an independent observer from the funding body at least annually and ad hoc as required to discuss trial issues.

#### Composition of the data monitoring committee, its role and reporting structure {21a}

The Data Monitoring and Safety Board (DSMB) consists of a chair (senior clinical trialist), two statisticians and a clinician. Interim data analyses are supplied, in strict confidence, to the DSMB, as frequently as the chair requests and meetings are held at least annually. The DSMB is independent of the trial organisers and sponsors. The terms of reference for the DMSB were agreed upon at their first meeting and documented in the DSMB charter. Based on interim data on the trial’s outcomes, adverse event data, accumulating evidence from other trials and any other relevant evidence, the DSMB will inform the Trial Steering Committee (TSC) if, in their view, there is proof beyond a reasonable doubt that the data indicate that any part of the protocol under investigation is either clearly indicated or contra-indicated, either for all trial participants or for a particular sub-group of trial participants. A difference of at least 3 standard errors in the interim analysis of a major endpoint may be needed to justify halting, or modifying, such a study prematurely.

#### Adverse event reporting and harms {22}

The processes of risk identification and management have been developed and tested. The trial has, in collaboration with the AHRI Clinical Research Department, identified and established referral pathways for the management of risk, including referrals to the local DoH and DSD services. The AHRI Clinical Research Department assists with the management of medical SAEs. We have developed a protocol for the management of these events and reporting at appropriate times to the DSMB.

The trial distinguishes between severe adverse events (SAEs) and adverse events (AEs). SAEs pose a high risk to the mother and/or enrolled child and require urgent attention and management. SAEs include maternal/child death; physical illness requiring hospitalisation ≥ 5 days; severe psychological or psychiatric illness (may require hospitalisation; current suicidal ideation with intention and/or a plan; self-harm; serious social harm (e.g. interpersonal violence-causing immediate danger or risk); stigma, emotional harm or risk of displacement/insecure housing (as a direct result of trial participation); and inadvertent disclosure of participants HIV status or breach of confidentiality (intentionally or unintentionally by research staff). Details of the SAE and a brief management plan are communicated to the DSMB Chair within 24 h of the team being notified of the SAE. Furthermore, SAEs are reported to the HSRC and OxTREC ethics committees within 7 days of the team being notified of the SAE.

In contrast, AEs are defined as events that do not pose an immediate risk to the mother/infant but require management and attention to prevent escalation into a high-risk event (SAE). These include relationship problems/conflicts, feelings of hopelessness and suicidal thoughts without serious intent or plans. These are monitored and recorded and reported to the ethics committees and the DSMB as AEs.

Additional information about the management of risk cases is documented under ‘Enhanced Standard of Care’ on page 23.

#### Frequency and plans for auditing trial conduct {23}

The trial is being conducted in accordance with the currently approved protocol, GCP, relevant regulations and SOPs. The risk management protocol and operational SOPs are reviewed as necessary throughout the trial to reflect significant changes to the protocol or outcomes of monitoring activities.

The AHRI research data management team conducts frequent checks of the trial, including recruitment patterns and the quality and completeness of data using relevant software as well as manual checks. Lists of missing data will be generated automatically for regular checks. Furthermore, the data collection software is designed to optimise correct data capture by specifying the data and values required.

#### Plans for communicating important protocol amendments to relevant parties (e.g. trial participants, ethical committees) {25}

Any protocol modifications which may influence the study conduct, potential benefit of the participants, participants’ safety, study objectives, study design, participant population, sample sizes, study procedures, interventions, assessments or significant administrative aspects will require a formal amendment to the protocol. Such amendment will be agreed upon by the Principal Investigators and the Investigators and submitted to OxTREC and HSRC for formal ethical approval before implementation. Participants will be informed of any important protocol amendments if deemed necessary.

### Authorship statement

The success of the trial depends upon a large number of collaborators. Credit for the trial findings will be given to all who have collaborated and participated in the trial including collaborators, members of the trial committees and trial staff. The authorship of the primary results paper arising will comprise all who have made a substantial intellectual contribution to the study (including the research question, design, analysis, interpretation), and so is expected to include all main applicants on this study who fulfil all four criteria of the ICMJE recommendations for authorship (www.icmje.org). The writing will be the responsibility of a writing committee including all of the investigators and core trial management team led by the Chief Investigator. All contributors to the trial will be listed at the end of the manuscript, with their contribution identified. It is the intention to publish at least two peer-reviewed articles detailing (i) the analysis of primary and secondary outcomes and (ii) the mechanisms by which the intervention improves infant outcomes will be evaluated in an exploratory analysis.

### Dissemination plans {31a}

The trial results will be disseminated to participants, the public, researchers, healthcare professionals and policymakers. The study participants will be informed of the findings from the trial. The trial results and their implications for policy and practice will be presented in the form of a technical brief to the district, provincial and national departments of health and disseminated nationally through webinars. The technical brief will also be disseminated to relevant mental health and public health charities and non-governmental organisations. With support from the AHRI community advisory board and public engagement office, we will disseminate the findings to the local community. The trial results will be presented at national and international conferences. Publications will be submitted to peer-reviewed, open-access journals in line with funder requirements.

## Discussion

There is a considerable treatment gap for mental health disorders amongst people living with HIV in LMICs [87], of which HIV-positive women in the perinatal period are considered especially vulnerable to poor mental health [3]. Addressing this treatment gap requires innovative programmes that can be integrated sustainably into existing primary care programmes with user involvement to create a holistic approach. Importantly, treating both maternal perinatal depression and enhancing child development has the potential to result in positive health and human capital benefits. If the integrated intervention is found to be effective, the ultimate aim is that it will be scalable at different levels, including local, provincial, national and international, and critically include users, stakeholders, the general public, policymakers and academic beneficiaries.

The intervention is potentially generalisable to countries with high levels of HIV as part of initiatives to prioritise antenatal and postnatal health care for mothers and children within fragile health systems.

The trial has developed an electronic treatment manual that guides the lay counsellors as they deliver BA and parenting support. There is a demand for improving the competencies and skills of the mental health and early childhood development workforce. Building capacity entails not only training frontline workers, many of whom are lay workers but contributing to an evidence-based body of knowledge that will be informative for future implementation strategies in community systems. The knowledge base will also contribute to supervision and training systems.

## Trial status

The trial started recruitment on 04/04/2018. Recruitment was suspended on 16/03/2020 due to the COVID-19 pandemic and was resumed briefly for 6 weeks in November and December 2020 but had to be paused in December 2020 due to further COVID-19 restrictions. Recruitment resumed again in March 2021 and was the closed on 13th of July 2021. The trial is currently operating on protocol version V1.05 (26th of May 2021 ).

## Abbreviations

AE: Adverse event
ART: Antiretroviral therapy
AHRI: Africa Health Research Institute
BA: Behavioural activation
BSID-III: Bayley Scales of Infant and Toddler Development III (BSID-III) cognitive subscale
CBCL: Child Behaviour Checklist Externalising Subscale
CBT: Cognitive behavioural therapy
CCD: Care for Child Development
CD4: Cluster of differentiation 4 (T cells or T-helper cells)
CHW: Community health worker
DALY: Disability-adjusted life years
DFID: Department for International Development, UK
DoH: Department of Health (South Africa)
DOI: Digital Object Identifier
DSD: Department of Social Development
DSMB: Data and Safety Monitoring Board
EBF: Exclusive breastfeeding
ECD: Early childhood development
EPDS: Edinburgh Postnatal Depression Scale
ESoC: Enhanced Standard of Care
GAD-7: Generalized Anxiety Disorder Scale 7-item
GCP: Good Clinical Practice
HIV: Human immunodeficiency virus
HSRC: Human Sciences Research Council, South Africa
ICC: Intra-cluster correlation coefficient
LMIC: Low- and middle-income country/ies
MICS: Multiple Indicator Cluster Surveys (Cognitive and Emotional Stimulation)
MRC: Medical Research Council
OxTREC: Oxford Tropical Research Ethics Committee
PI: Principal Investigator
PSI/SF: Parenting Stress Index Short Form
RCT: Randomised controlled trial
REC: Research Ethics Committee
SAE: Serious adverse event
SD: Standard deviation
SoC: Standard of care
TSC: Trial Steering Committee
UKRI: UK Research and Innovation
UNICEF: United Nations International Children’s Emergency Fund
VL: Viral load
WHO: World Health Organization

## Footnotes

1 ‘End of pregnancy’ refers to ~ 35 weeks of gestation in this paper.

2 Episodes of diarrhoea are defined as maternal report of diarrhoea in the previous 14 days [52]
